# A Proof-of-Mechanism Study to Test Effects of the NMDA Receptor Antagonist Lanicemine on Behavioral Sensitization in Individuals With Symptoms of PTSD

**DOI:** 10.3389/fpsyt.2019.00846

**Published:** 2019-12-13

**Authors:** Marijn Lijffijt, Charles E. Green, Nicholas Balderston, Tabish Iqbal, Megan Atkinson, Brittany Vo-Le, Bylinda Vo-Le, Brittany O’Brien, Christian Grillon, Alan C. Swann, Sanjay J. Mathew

**Affiliations:** ^1^Research Service Line, Michael E. DeBakey VA Medical Center, Houston, TX, United States; ^2^Menninger Department of Psychiatry and Behavioral Sciences, Baylor College of Medicine, Houston, TX, United States; ^3^Department of Psychiatry and Behavioral Sciences, UTHealth McGovern Medical School, University of Texas Health Science Center at Houston, Houston, TX, United States; ^4^Department of Pediatrics - Center for Evidence Based Medicine, UTHealth McGovern Medical School, University of Texas Health Science Center at Houston, Houston, TX, United States; ^5^Section on Neurobiology of Fear and Anxiety, National Institute of Mental Health, National Institutes of Health, Bethesda, MD, United States; ^6^Department of Anesthesiology, Michael E. DeBakey VA Medical Center, Houston, TX, United States; ^7^Department of Anesthesiology, Baylor College of Medicine, Houston, TX, United States; ^8^Mental Health Care Line, Michael E. DeBakey VA Medical Center, Houston, TX, United States

**Keywords:** behavioral sensitization, NMDA receptor, hyperarousal, neurophysiology, post-traumatic stress disorder, anxiety potentiated startle

## Abstract

**Background:** Individuals with post-traumatic stress disorder (PTSD) have a heightened sensitivity to subsequent stressors, addictive drugs, and symptom recurrence, a form of behavioral sensitization. N-methyl-D-aspartate receptors (NMDARs) are involved in the establishment and activation of sensitized behavior.

**Objective:** We describe a protocol of a randomized placebo-controlled Phase 1b proof-of-mechanism trial to examine target engagement, safety, tolerability, and possible efficacy of the NMDAR antagonist lanicemine in individuals with symptoms of PTSD (Clinician Administered PTSD Scale [CAPS-5] score ≥ 25) and evidence of behavioral sensitization measured as enhanced anxiety-potentiated startle (APS; T-score ≥ 2.8).

**Methods:** Subjects (n = 24; age range 21–65) receive three 60-min intravenous infusions of placebo or 100 mg lanicemine over 5 non-consecutive days. Primary endpoint is change in APS from pre-treatment baseline to after the third infusion. NMDAR engagement is probed with resting state EEG gamma band power, 40 Hz auditory steady state response, the mismatch negativity amplitude, and P50 sensory gating. Change in CAPS-5 scores is an exploratory clinical endpoint. Bayesian statistical methods will evaluate endpoints to determine suitability of this agent for further study.

**Conclusion:** In contrast to traditional early-phase trials that use symptom severity to track treatment efficacy, this study tracks engagement of the study drug on expression of behavioral sensitization, a functional mechanism likely to cut across disorders. This experimental therapeutics design is consistent with recent NIMH-industry collaborative studies, and could serve as a template for testing novel pharmacological agents in psychiatry.

**Clinical Trial Registration:** www.ClinicalTrials.gov, identifier NCT03166501.

## Introduction

With few exceptions, the explosion in basic neuroscience knowledge over the past two decades has not led to the development of mechanistically novel treatments for serious psychiatric disorders. It has been argued that the traditional approach to psychiatric clinical trials, which solely rely on symptom rating scales as endpoints, has stymied progress. To address this problem, the United States National Institute of Mental Health (NIMH) has adopted an experimental therapeutics approach to early-phase clinical trials, which require proof-of-mechanism (POM) studies to determine whether an experimental intervention is viable for testing in larger randomized controlled trials (www.nimh.nih.gov/about/strategic-planning-reports/strategic-research-priorities/index.shtml). The experimental therapeutics approach mandates (i) a functional mechanism that is likely to be associated with both clinical phenotype and therapeutic response, (ii) an intervention that is likely to act on a target that engages the functional mechanism [target specificity], (iii) a biomarker to show that the intervention engages the hypothesized target [target engagement], and (iv) a biomarker to show that the intervention engages the hypothesized functional mechanism [POM] (1, 2). Selection of the functional mechanism can be guided by Research Domain Criteria (RDoC). RDoC regards psychiatric disorders not as DSM-defined clinical phenotypes but as disease-overlapping functional domains associated with neuroscience-inspired neural circuitries that are at the crossroads of genotype and clinical phenotype (3, 4).

In this paper we describe the rationale and protocol for an NIMH-funded POM study to determine whether the N-methyl-D-aspartate receptor (NMDAR) antagonist lanicemine (BHV-5500) engages the NMDAR to block expression of behavioral sensitization underlying symptoms of post-traumatic stress disorder (PTSD).

## Post-Traumatic Stress Disorder

PTSD is a trauma-induced condition marked by intrusive thoughts about and re-experiencing of trauma (life-like flashbacks, nightmares), avoidance of trauma-associated stimuli and thoughts, negative cognitions and mood (anhedonia, hopelessness), and hyperarousal (anger outbursts, hypervigilance, exaggerated startle) (5). An estimated 6% of the U.S. population has a lifetime history of PTSD (6, 7), with a higher prevalence among first responders (8), military veterans (9–12), and women (6, 7, 12). An additional 6% of U.S. adults could have a lifetime history of partial PTSD defined as fewer, but not less frequent, intense or severe, PTSD symptoms (13). Mean age of PTSD onset is 24 (7), although about half of the individuals with lifetime PTSD meet criteria before age 18 (6). While there are several evidence-based psychotherapies for PTSD, there remain only two U.S. Food and Drug Administration (FDA)-approved drugs for PTSD, the selective serotonin reuptake inhibitors paroxetine and sertraline. Both of these approved medications have only low to moderate efficacy for improving PTSD symptoms (14). This highlights the importance of finding novel targets for intervention.

## Functional Mechanism

Behavioral sensitization refers to a process whereby trauma-associated stress (but also repeated use of substances of abuse, mood or anxiety episodes, and suicide attempts) sensitize behavioral, motivational and stress systems, thereby increasing the behavioral and physiological reactivity to subsequent stressors or other sensitizing agents even after a prolonged absence of those agents (15–18). Consistent with findings in animals, research in humans showed at least three different aspects of behavioral sensitization: *induction*, the development of behavioral sensitization to a sensitizing agent, including uncontrollable stressors (19), substances of abuse (18, 20, 21), and, in PTSD, repeated illness episodes (22); *expression*, exaggerated behavioral or physiological responses to a sensitizing agent even after prolonged absence of that agent (18, 21, 23); and c*ross-sensitization*, the process by which sensitization to one agent results in sensitization to other agents (e.g., facilitation of behavioral sensitization to psychostimulants after exposure to uncontrollable stress) (24). Animal research showed that all three aspects of behavioral sensitization require activation of NMDARs albeit *via* different neural pathways. Repeated cocaine administration increased NMDAR sensitivity only in rats that developed a sensitized motor response (25). Blockade of NMDARs by non-competitive NMDAR antagonist ketamine or MK-801 prevented *induction* of behavioral sensitization to ethanol (26, 27), apomorphine (28), stimulants (29–36), stress (36), and nicotine (37, 38), blocked *expression* of behavioral sensitization to alcohol (39), stimulants (29–32, 35, 40), stress (41), and nicotine (38), and blocked *cross-sensitization* between ethanol and stimulants (40, 42) and between stress and stimulants (36, 43, 44). Administration of MK-801 in the nucleus accumbens prevented induction but not expression of behavioral sensitization to stimulant administration (45), consistent with involvement of different neural pathways in induction and expression of behavioral sensitization.

Our premise for this study is that PTSD symptoms are associated with behavioral sensitization (46–48). We further propose that this type of sensitization could be blocked rapidly with an NMDAR antagonist (36, 43, 44) to relieve PTSD symptoms.

## Primary Outcome Measure: Biomarker of Functional Mechanism

Behavioral sensitization in PTSD is associated with hyperarousal of the extended amygdala—the basolateral amygdala (BLA), central amygdala, medial amygdala, bed nucleus of the stria terminalis (BNST), shell of the nucleus accumbens, and their interconnectivity (48, 49). In animals, uncontrollable stressors, including trauma, enhanced sensitivity of the extended amygdala to future, milder, stressors (19, 50). In humans, enhanced reactivity of the amygdala could predispose to development of PTSD symptoms (51, 52) and is associated with less resilience to stressors of everyday life (53). Sensitization of the extended amygdala cuts across all RDoC functional domains (49) and has been associated with most PTSD symptom clusters (48).

NMDARs antagonists may improve PTSD symptoms by affecting behavioral sensitization. In preclinical models, NMDAR antagonists blocked induction (36) and expression (41) of behavioral sensitization by stress, and blocked cross-sensitization between stress and stimulants (36, 43, 44). In humans, intraoperative administration of NMDAR antagonist ketamine may reduce PTSD risk (54, 55), and a single infusion of 0.5 mg/kg ketamine compared to midazolam in patients with PTSD resulted in rapid (within 24 h) and sustained (at least 7 days) improvement in PTSD symptoms (56, 57). Finally, the low-affinity NMDAR antagonist memantine improved hyperarousal and depressive symptoms in individuals with PTSD (58).

In this study we operationalize expression of behavioral sensitization as an aversion-potentiated startle amplitude expressed as T-score obtained on the No-threat, Predictable-threat, Unpredictable-threat (NPU) test (59–61). Sudden intense stimuli elicit an eye blink startle reflex that can be potentiated in negative emotional states (62, 63). Phasic negative emotional states potentiate startle *via* activation of the central amygdala in response to an explicit cue that signals threat of an uncontrollable aversive stimulus, assessing fear [fear-potentiated startle (FPS)]. Tonic negative emotional states potentiate startle primarily *via* activation of the BNST in response to a context, not a specific cue, that signals threat of an uncontrollable aversive stimulus, assessing anxiety [anxiety-potentiated startle (APS)] (62, 63). PTSD is associated with an enhanced APS but not FPS (64, 65), which could be related to increased activation of BNST excitatory glutamatergic neurons relative to BNST inhibitory GABA neurons (66) or with decreased regulation of the prefrontal cortex over the amygdala (67) associated with diminished prefrontal glutamate concentration (68).

We propose a POM study using lanicemine 100 mg in patients with symptoms of PTSD with evidence of behavioral sensitization operationalized as an enhanced APS. Using air puffs to the forehead as startle probes and loud acoustic sounds as aversive stimuli, the NPU-threat test discriminated between patients with PTSD (n = 16; APS T-score mean ± SD = 6.5 ± 1.4) and healthy controls (n = 34; APS T-score = 0.6 ± 1.1) (65). For this trial, we select participants with at least moderate symptoms of PTSD operationalized as a CAPS-5 score ≥25, and a state of behavioral sensitization operationalized as an APS T-score at least two standard deviations above the mean among healthy volunteers (APS T-score ≥2.8). We anticipate approximately 60% of PTSD patients to meet APS entry criteria (Grillon, unpublished data). The primary endpoint of this study is change in APS T-score from pre-treatment baseline to that following the last of three lanicemine infusions. Paradigm specifics are described in **Supplemental Materials**.

## Experimental Drug

Lanicemine is an intravenously administered NMDAR antagonist that crosses the blood-brain barrier, binding within channel pores (Ki 0.5–3 µM) of NR2A and NR2B NMDAR complexes (IC_50_ 4–40 µM) to block the flow of charged cations. In contrast to other NMDAR antagonists such as ketamine and MK-801, lanicemine has a fast off-rate and is low-trapping, properties associated with favorable safety and tolerability profiles (69). In addition, ketamine’s actions include effects on systems beyond NMDAR channel blockade, including at opiate, sigma, and muscarinic receptors (70), whereas lanicemine has negligible off-target pharmacological effects and provides a more selective NMDAR probe. Lanicemine has been examined in preclinical and early-phase clinical studies in patients with stroke, sleep apnea, and treatment-resistant depression (TRD) and is considered safe in humans. A recently completed phase 2b trial found that lanicemine 100 mg dose was effective in individuals with TRD who had the most severe depression or suicidal ideation at the start of the study (71), potentially suggestive of effects on behavioral sensitization.

Prior studies exposed healthy individuals and patients with treatment resistant depression to single or repeated infusions of lanicemine 50 mg or 100 mg (69, 71, 72). At least one adverse event (AE) was reported by 77.1% of subject in the lanicemine arms, compared to 70% of subjects in the placebo arm. Although most AEs were of mild or moderate intensity, a greater proportion of subjects discontinued the trials due to an AE for lanicemine 100 mg (9.0%) than lanicemine 50 mg (2.0%) or placebo (4.0%). Dizziness was the most common AE and appeared to be dose-related. Other reported side effects included: changes in balance, feeling drunk, blurred vision, headache, sleepiness, weakness, impaired concentration, abnormal sensations (tingling of hands, feet, feelings of crawling ants within body), nausea, and vomiting. Lanicemine has been associated also with dose-dependent transient mild elevations in blood pressure with no evidence of sustained changes in blood pressure or pulse rate. There were no clinically meaningful differences between lanicemine and placebo groups for mean changes in clinical chemistry, hematology, or urinalysis. Treatment with lanicemine was not associated with any decline in psychomotor function, attention, working memory, learning, or general cognitive function. An important difference between lanicemine and ketamine concerns dissociative side effects. Although 8% of patients with treatment resistant depression in the lanicemine 100 mg arm spontaneously reported dissociative symptoms compared to 4% in both the lanicemine 50 mg and placebo arms, only 1.1% of patients in both lanicemine groups had a Clinician-Administered Dissociative States Scale (CADSS) score classified as high (11–25) at any time point. In contrast, 50% of patients with treatment resistant depression showed high dissociation with a 40 minute subanesthetic infusion of ketamine (73).

## Secondary Outcome Measures

### Biomarkers of NMDAR Target Engagement

NMDAR target engagement is examined with neurophysiological measures that are translatable between animal and human studies.

Subanesthetic doses of NMDAR antagonist ketamine increased resting-state gamma band (30–100 Hz) power in animals (74–76) and humans as a function of time since start of ketamine infusion, rapidly normalizing after end of infusion (69, 77, 78). This response has been associated with activation of α-amino-3-hydroxy-5-methyl-4-isoxazolepropionic acid receptors (AMPAR) (79) or activation of fast-spiking gamma-aminobutyric acid (GABA)-ergic parvalbumin interneurons (80) following a ketamine-driven increase in prefrontal glutamate (81). Lanicemine 75 mg and 150 mg increased midline electrodes gamma band activity in a dose-response fashion in healthy volunteers (69). This increase was unrelated to increased motor activity, separating it from ketamine-increased gamma band power that did correlate with enhanced motor activity (69). Ketamine-increased gamma band power was unrelated to treatment response in patients with TRD (78). We hypothesize that resting state gamma band power will increase with lanicemine 100 mg compared to placebo.

The 40 Hz auditory steady state response (40 Hz ASSR) measures stimulus-evoked gamma band (40 Hz) power and phase synchronization. In awake rats, a single intravenous bolus of 1, 3, 10, and 30 mg/kg ketamine increased intracranial frontocentral 40 Hz power, but the time was delayed or duration prolonged linearly with increase in dose (82). Phase synchrony decreased at the lowest dose and increased at the highest dose, with changes correlating negatively with ketamine NMDAR occupancy (82). A single dose of MK-801 increased intracranial 40 Hz phase synchrony but did not affect power (83). In healthy humans and humans with schizophrenia, memantine 20 mg increased frontal 40 Hz power and phase locking; memantine 10 mg had no effects (84). We expect lanicemine 100 mg to increase 40 Hz ASSR power and phase synchrony.

The auditory Mismatch Negativity (MMN) is evoked between 100 and 250 ms after an unexpected auditory event, and is measured as the difference between (frequent) expected and (infrequent) unexpected stimuli in a passive oddball task (85). MMN amplitude could be increased in PTSD (64, 86, 87), although this has not been found uniformly (88). MMN in healthy volunteers was increased under threat of an aversive stimulus (89) suggesting it could be sensitive to behavioral sensitization. Ketamine suppressed MMN amplitude (90–92), perhaps *via* blockage of NR2B receptor units (93), without affecting temporally overlapping ERP components (92). This effect is sustained for at least 30 min after end of infusion (92). Memantine could increase MMN amplitude (94). MMN amplitude is included as a measure of target engagement as well as a measure associated with functional mechanism. We expect suppression of MMN amplitude with lanicemine compared to placebo, potentially associated with change in APS.

P50 auditory sensory gating measures P50 amplitude suppression following presentation of the same auditory stimulus in short temporal proximity, reflecting pre-attentional information filtering (95–98). P50 gating is impaired in PTSD compared to trauma controls (99), and after acute stress in healthy individuals (100, 101). Lanicemine may normalize P50 gating if lanicemine attenuates stress reactivity. However, ketamine 0.3 mg/kg (bolus) in healthy volunteers impaired P50 gating through ketamine-induced increases in gamma band activity for the second stimulus of the repetition (102) which was also found in rats (76). Thus, lanicemine may normalize or worsen P50 gating. We study effects of lanicemine on P50 gating to examine this apparent discrepancy and in order to characterize its effects on aspects of information filtering.

Further specifics of the paradigms can be found in **Supplemental Materials**.

### Symptom Severity Rating Scales

The Clinician Administered PTSD Scale (CAPS-5) (103) is a structured interview to assess intensity and frequency of DSM-5 PTSD symptoms. The CAPS-5 is administered at screening to determine subject eligibility (104), before the first infusion, and 3 days after the last infusion (day 8) to preliminarily examine treatment efficacy (105). Further specifics of the CAPS-5 and of other clinical measures can be found in the accompanying **Supplemental Materials**.

## Sample Size

The primary statistical contrast is lanicemine compared to placebo on APS from baseline to the end of the third infusion. Assuming an observed posterior probability of at least 0.75 of Cohen’s d < -0.4 in the active condition relative to placebo and a correlation r = 0.5 due to repeated measures, K = 500 Monte Carlo simulations indicated that a sample size of 20 allows an 82% chance of detecting superiority of lanicemine. Allowing for attrition, we will recruit 24 subjects. To date, we randomized 23 of the 24 subjects.

## Study Design

This study is funded by the NIMH under an R61/R33 Phased Innovation Award (5R61MH110540). Biohaven Pharmaceutical Holding Company LTD provided material support. The study drug is administered as an intravenous solution under FDA IND number 134304; the trial is registered at ClinicalTrials.gov under NCT03166501. All study-related procedures and materials have been approved by the Baylor College of Medicine Institutional Review Board, and the MEDVAMC Office of Research and Development. An NIMH-appointed data and safety monitoring board (DSMB) provides oversight.

This study uses a randomized, double-blind, parallel-arm, placebo-controlled fixed dose design to test lanicemine (100 mg) compared to saline placebo in up to 24 male and female outpatients between the ages of 21 and 65 who have significant PTSD symptoms (CAPS-5 score of at least 25, and a Clinical Global Impression of Severity [CGI-S] score of at least 4), and physiological manifestations of behavioral sensitization (APS T-score of at least 2.8). Inclusion and exclusion criteria are displayed in **Supplemental Material**.

To qualify for randomization, entry criteria must be met at screening and the morning of the first lanicemine infusion. Subjects are randomized in a 1:1 ratio to receive three 60-min intravenous infusions of lanicemine or placebo on non-consecutive days over a 5-day period. **Table 1** provides the timeline of all study procedures. **Table 2** provides the timeline of study procedures at the first infusion (day 1) and third infusion (day 5).

**Table 1:** Schedule of events.

	Screening^a^	Treatment period^b^			Telephone follow-up	EOS/ET^c^
	Day -42 to -1	Day 1 (1^st^ infusion)	Day 3 (2^nd^ infusion)	Day 5 (3^rd^ infusion)	Day 8 ± 2	Day 19 ± 4
Informed consent	X					
Eligibility criteria	X	X				
Demographics	X					
Psychiatric and family history	X					
Medication history	X	X	X	X	X	
Medical history	X					
Alcohol and drug history	X					
MINI	X					
CAPS-5	X	X			X	X
PCL-5	X	X			X	X
CGI-S^d^	X	X	X	X	X	X
CGI-I^d^	X	X	X	X	X	X
C-SSRS^d^	X	X	X	X	X	X
Vital signs (supine)^e^	X	X	X	X		X
Orthostatic BP^f^	X	X	X	X		
Physical examination	X	X				X
Weight, height, BMI	X	X				
Digital 12-lead ECG^g^	X	X	X	X		X
Metabolic panel	X	X				X
HgbA1c	X					
Serum pregnancy test	X					X
Urine pregnancy test^h^		X	X	X		
Urinalysis	X	X				X
Urine drug screen	X	X	X	X		X
Adverse events	X	X	X	X	X	X
PK sampling		X		X		
NPU-threat test		X		X		
Mismatch negativity (MMN)		X		X		
Resting state EEG		X		X		
40 Hz ASSR		X		X		
P50 auditory sensory gating		X		X		

^a^Screening visit can be completed over 2–3 days; ^b^Infusions must occur on non-consecutive days within a 6 day maximum period; ^c^End of study (EOS)/early termination (ET) visit conducted at the time of discontinuation; ^d^Measures administered prior to any infusion; ^e^Measured at time 0 (within 1 h before infusion is acceptable) and at the end of infusion; ^f^Orthostatic blood pressure (BP) will be measured at time 0 (within 1 h before infusion is acceptable) and either at least 1 or 3 h after the end of infusion; ^g^Measured at time 0 (within 1 h before infusion is acceptable) and at the end of each infusion; ^h^A negative result is required prior to each infusion.

**Table 2:** Study procedures during infusion 1 (day 1) and infusion 3 (day 5) ^a^.

	Time (hours from start of infusion)					
	*T0* (-3)	*T1* (-1.5)	*T2* (+0.5)	*T3* (+1.5)	*T4* (+3.5)	*T5* (+4)
Urine pregnancy test	X					
CAPS-5	X ^b^					
NPU-threat test	X					X
MMN		X	X	X	X	
Resting state EEG		X	X	X	X	
40 Hz ASSR		X	X	X	X	
P50 auditory sensory gating		X		X	X	
PK sample		X	X	X	X	X

^a^Order of procedures is fixed; ^b^CAPS-5 is assessed only before infusion 1.

## Data Analysis Plan

### Primary Endpoint

We expect that relative to placebo, three infusions of lanicemine will normalize the APS response after the last infusion. The primary analysis for this endpoint is regression of the APS measured on the fifth day of treatment (third infusion) onto treatment group after controlling for APS measured at baseline of the first day of treatment (first infusion).

### Secondary Endpoints

We expect that relative to placebo, lanicemine will demonstrate effects on neurophysiology measured on the fifth day of treatment, and on the CAPS-5 measured 3 days after the last infusion (day 8). The primary analyses for these endpoints are the regression of neurophysiology measures, measured after infusion 3, and CAPS-5, measured on day 8, onto treatment group after covariation for the respective baseline values. Secondary analyses will use multilevel models to evaluate changes as a function of stratification variables, treatment, time and the interaction of treatment and time. In addition, Bayesian Structural Equation Modeling will be used to test the hypothesis that treatment effects on day 5 APS or neurophysiology mediate treatment effects on day 8 CAPS-5.

### Analysis Sets

Analyses will be performed on a:

Modified intent-to-treat (mITT) analysis set: this analysis set will include all randomized patients who took study medication, and who have a baseline APS and at least 1 post-baseline behavioral rating. The mITT analysis set will be used for exploratory efficacy analyses.

Per-protocol (PP) analysis set: This analysis set will include only those mITT patients without significant protocol deviation, and who received the treatment to which they were randomized. The analysis of the primary efficacy variable will be repeated on this set, and additional analyses of efficacy variables may be performed using this set to assess the robustness of the treatment effects.

Safety analysis set: This analysis set will include all randomized patients who were given at least one dose of study medication, on whom any post-dose data are available, and who are classified according to the treatment actually received (i.e., erroneously treated patients will be accounted for in their actual treatment group). This set will be used to assess safety and tolerability variables.

### Specific Analytic Strategies

Efficacy and safety variables will be summarized using descriptive statistics and graphs. Continuous variables will be summarized by descriptive statistics. Categorical variables will be summarized in frequency tables. Pharmacokinetic variables will be summarized by the geometric mean and coefficient of variation. Lanicemine will be compared with placebo. Where appropriate, we will report model-based point estimates together with their 95% credible intervals.

Preliminary data analyses examining group differences for demographic and baseline variables will use cross-tabulation, ANOVA’s, and examination of correlations between baseline variables and specified outcomes. For the purposes of evaluating the comparability of groups, posterior probabilities of ≥95% will constitute evidence for statistically reliable differences. Baseline or demographic variables on which group differences are detected, and which are correlated with outcomes meet the definition of confounders (106, 107), and will result in two sets of analyses: one in which the relevant variable is included as a covariate and one in which it is not. This will permit determination of the degree to which any covariate might confound conclusions regarding treatment. The data analytic strategy will use generalized linear modeling (GLM) and multilevel models for both continuous and discrete outcomes. Cross-sectional continuous, count, dichotomous and time to event data will be evaluated using GLM and proportional hazards regression, respectively. All analyses will be conducted on a mITT analysis set.

Bayesian approaches will implement joint modeling of observed outcomes and the missing data which is robust to ignorable missingness (108). Sensitivity analyses will evaluate robustness of analytic conclusions to missing data. Non-ignorable missing data patterns will be addressed through pattern-mixture modeling methods (109). Evaluation of posterior distributions will permit statements regarding the probability that effects of varying magnitudes exist, given the data. Specification of diffuse, neutral priors will reflect the initial uncertainty regarding effect sizes. For all generalized linear mixed models, priors for regression coefficients will be specified as ∼Normal (µ = 0, σ^2^ = 1 x 10^6^); level one error variances will be specified as ∼Folded t-distribution (ν = 3, µ = 0, σ = 1000). Prior distributions for level-two variances in multilevel models will follow ∼Folded t-distribution (ν = 3, µ = 0, σ = 1000). Priors for the comparison of proportions will be specified as ∼Beta (α = 0.5, β = 0.5). Sensitivity analysis using optimistic and pessimistic, skeptical priors will evaluate prior assumptions (110). Decisions regarding the degree to which treatment confers benefit, and whether further confirmatory trials are warranted will be based on the posterior distribution of effect sizes. If the observed posterior probability of a Cohen’s d < -0.4 is at least 0.75, this will be sufficient evidence to proceed to a larger POC clinical trial.

Assessing the convergence of Bayesian analyses on the posterior distributions *via* Monte-Carlo Markov chain (MCMC) will use graphical (Trace Plot, Autocorrelation Plot) and quantitative (Gelman-Rubin Diagnostics and Effective Sample Size) evidence. Mediational modeling will permit estimates of the indirect effects of treatment on primary and secondary endpoints using the product coefficient method (111). Bayesian Structural Equation Modelling (BSEM) prior specification will adapt recommendations from Muthén and Asparouhov (112). A Bayesian estimate for the indirect effect employs the posterior distribution of the parameter (i.e. the product coefficient): a density denoting the probability that the different values of the parameter obtain given the observed data. This posterior distribution may be further partitioned to evaluate the probability that the true parameter falls within a specific range of values. This will facilitate decision-making regarding the relative merits of continued investigation of the compound. Use of the MCMC approach in Bayesian analyses has demonstrated superiority for small sample performance compared to maximum likelihood-based approaches in continuous, normally distributed data (111). These properties are likely due to the MCMC approach’s lack of reliance on large sample size assumptions (113). Sensitivity analysis using optimistic and pessimistic, skeptical priors evaluates prior assumptions (110).

## Conclusion

This POM clinical trial examines a novel target—trauma-induced behavioral sensitization—hypothesized to be associated with PTSD symptoms. The expression of behavioral sensitization is measured as an exaggerated APS reflecting enhanced reactivity of the BNST, a component of the extended amygdala, to uncontrollable stressors. Behavioral sensitization in general, and BNST reactivity specifically, may be under the control of NMDARs (66). Lanicemine potentially addresses the interaction between behavioral sensitization and acute behavioral and cognitive disturbances that characterize PTSD through its interaction with NMDAR that we will test using neurophysiological measures sensitive to NMDAR agents. If lanicemine engages the functional target and is safe and efficacious in this difficult-to-treat population, there is genuine translational potential for this compound or similar agents in treatment of PTSD and of other disorders potentially involving behavioral sensitization, such as bipolar disorder or substance use disorders. This study does have limitations that limit the generalizability of outcomes. The outcomes are limited to individuals with high APS. In addition, we exclude individuals with disorders that frequently co-exist with PTSD, and with medications that are frequently used for PTSD. Finally, studies with lanicemine and placebo in individuals with treatment resistant depression have revealed a more pronounced placebo response for individuals with fewer symptoms of depression, absent or milder suicidal ideation, and no treatment with antipsychotic medication (71). Our decision to enroll individuals with symptoms of PTSD instead of individuals who meet full PTSD criteria may benefit the placebo arm over the lanicemine arm. However, the findings regarding placebo apply to effects on symptoms of depression and may not generalize to symptoms of PTSD, and findings may not generalize to biomarkers of functional mechanisms or to biomarkers of target engagement.

This study is funded under an R61/R33 Phased Innovation Award (R61 MH10540-01). This award consists of an R61 POM study followed by an R33 phase to test clinical efficacy of a study drug. The R33 study would only commence if the POM study shows evidence of engagement by lanicemine of the functional mechanism (change in APS) and the biological target (change in neurophysiological measures). The go/no-go framework for the R61 study is operationalized such that if there is significant symptom improvement without a clear effect of functional mechanism and evidence of target engagement, the drug would not be tested further in an R33. A potential risk of this approach is that the measures of functional mechanism and of target engagement selected may not be appropriate even if the drug does engage the proposed mechanisms. This approach is in marked contrast to usual industry-supported early phase trials in which early signals of clinical efficacy drive the decision to move forward with larger Phase 2 studies, irrespective of information gleaned from biomarkers.

In conclusion, this study tracks engagement of the study drug on a functional mechanism likely to cut across disorders, which is consistent with recent NIMH-industry collaborative studies (114) and “Fast-Fail” trials (2, 115), and could serve as a template for testing pharmacological agents in psychiatry.

## Ethics Statement

The study drug is administered as an intravenous solution under FDA IND number 134304; the trial is registered at ClinicalTrials.gov under NCT03166501. All study-related procedures and materials have been approved by the Baylor College of Medicine Institutional Review Board, and the MEDVAMC Office of Research and Development. An NIMH-appointed data and safety monitoring board (DSMB) provides oversight.

## Author Contributions

SJM, ACS, CEG, and ML conceived and developed the project. ML, NB, TI, MA, BrV-L, By V-L, BOB, CG, ACS, and SJM contributed to implementing the project. ML, CEG, and SJM wrote the draft version and the final version of this manuscript. NB, TI, MA, BrV-L, ByV-L, BOB, CG, and ACS contributed to the draft version and the final version of this manuscript.

## Funding

This work was supported by the National Institute of Mental Health through grant 5R61MH110540. The views expressed in this publication do not necessarily reflect the official policies of the U.S. Department of Veterans Affairs.

## Conflict of Interest

SM is PI on a Biohaven-sponsored clinical trial.

The remaining authors declare that the research was conducted in the absence of any commercial or financial relationships that could be construed as a potential conflict of interest.

The authors declare that this study received material support from Biohaven Pharmaceuticals. Biohaven Pharmaceuticals was not involved in the study design, data collection, data analysis, interpretation of data, the writing of this article or the decision to submit it for publication.
