# Supplementary material for: A Proof-of-Mechanism Study to Test Effects of the NMDA Receptor Antagonist Lanicemine on Behavioral Sensitization in Individuals With Symptoms of PTSD
Source: Front Psychiatry. 2019 Dec 13;10:846. doi: 10.3389/fpsyt.2019.00846 (PMC6923195; doi:10.3389/fpsyt.2019.00846)
Supplement: Supplementary file 1 [file DataSheet_1.pdf]

SUPPLEMENTAL MATERIALS

PROCEDURES

Primary Outcome Measures: NPU-threat test

APS at screening will be used to determine subject eligibility, and APS before the first infusion and 4 hours after the last infusion will be used to determine efficacy. The test consists of three conditions: neutral (N) without chance of an aversive stimulus, predictable (P) with a chance of an aversive stimulus during presentation of a cue but not during a cue-free period (ITI), and unpredictable (U) with a chance of an aversive stimulus at any time (cue and ITI) (1). Conditions are designated by colored shapes with a short description at the top of the screen. Subjects are verbally instructed before the start of the test about cue – aversive stimulus contingencies. We adapted the NPU-threat test that previously differentiated people with PTSD from people with generalized anxiety disorder and healthy controls, with healthy controls showing only marginal APS (2).

APS is measured with the NPU-threat test. The startle probe is a 40-ms, 40-psi air puffs to the forehead one to two cm above the bridge of the nose, avoiding leakage of air towards the eye (The 7-psi air puff in the original task did not elicit consistent startle reactions). Aversive stimuli are 3-sec 95-dB white noise, 1kHz pure tone and 1.2kHz pure tone randomized across conditions P and U (the original task used 4 aversive stimuli: white noise, a fire alarm, a 1kHz pure tone, and a female scream accompanied by an image of a scared woman; due to software constraints we use 3 aversive stimuli).

Testing was preceded by one habituation block of 10 startle probes. Order of within-subject condition presentation is NPNUN-NUNPN or NUNPN-NPNUN; presentation was divided into two 10-min blocks due to software constraints. Each block started with 4 startle habituation trials. Each N, P, and U condition has 5 cue-ITI pairs, with two startle probes. Instead of four trials per condition and 6 startle probes (3 during a cue and 3 during ITI), we use five and 8 startle probes to enhance startle signal reliability (3) and which is consistent with current guidelines (1). Cues are presented for 10s; ITI is 14 to 24-ms. Startle probes are presented 5-s to 7-s after cue onset; probe-probe interval is 18-s to 24-s. Aversive stimuli are delivered on 2 P and 2 U trials per block. No startle stimulus was delivered within 8 sec of an aversive stimulus. The aversive stimulus was presented after the startle probe in the P condition, and was presented at least 8 seconds before a startle probe in the U condition (1).

Secondary Outcome Measures: Electrophysiology

Electrophysiology is collected with Curry 7.0.10 software using a SynAmps-RT 64-channel amplifier (Compumedics Neuroscan, Charlotte, NC, USA) and a 64-channel actiCAP (Brain Vision, Morrisville, NC, USA). Continuous data is collected at 1000 kHz with a gain of 10, and filtered between DC and 400 Hz. During testing, subjects rest on a bed in a 50-70 degree supine position. Before each task, subjects are instructed about the task, are reminded to minimize general movements and eye movements, to relax the neck and face muscles, and to keep eyes open (except for eyes-closed EEG); blinking is allowed. Because of the setting in a hospital, the room is not protected from electrical and other noise.

Resting state EEG gamma band power is obtained from two sets of one minute eyes closed and one minute eyes open.

The MMN amplitude is measured with a passive oddball task following recommended guidelines (4). The task consists of 557 50-ms, 80-dB SPL, 1kHz standard tones (90%) and 63 100-ms, 80-dB SPL, 1kHz deviant tones (10%). Each deviant is preceded by 5-14 standards. Interstimulus interval is 500ms.

The 40Hz ASSR consisted of 100 1-sec trains of 40 1ms-duration, 80dB 1kHz carrier frequency clicks. Interstimulus interval is 500ms. To ensure vigilance, subjects count the ten 2kHz click trains.

P50 sensory gating is measured with a paired-click paradigm (5,6). The task consists of 40 S1-S2 pairs of identical 4ms, 80dB SPL, 1kHz clicks. Inter-click interval is 500 ms; inter-pair interval is 5-8s.

Secondary Outcome Measures: Patient or Clinician Symptom Severity Rating Scales

Life Events Checklist for DSM-5 (LEC-5) (7) is a self-report measure used along with the CAPS-5 to screen for potentially traumatic events in a respondent's lifetime. It assesses exposure to 16 events known to potentially result in PTSD or distress and includes one additional item assessing any other extraordinarily stressful event not captured in the first 16 items.

The **Clinician Administered PTSD Scale** (CAPS-5) (8) is a structured interview that corresponds to the DSM-5 criteria for PTSD. The full CAPS-5 is assessed after a LEC-5 and CAPS-5 identified index trauma event. The CAPS-5 is used to make a current (past month) (9) diagnosis of PTSD for subject eligibility, or to assess symptoms over the past week (10) to determine baseline PTSD symptom severity before infusion 1 and to determine treatment effect at day 8. In addition to assessing the core PTSD symptoms, questions target the impact of symptoms on social and occupational functioning, improvement in symptoms since a previous CAPS-5 administration, overall response validity, overall PTSD severity, and associated symptoms (depersonalization and derealization).

The **Clinical Global Impression of Severity and Improvement** (CGI-S, CGI-I) (11) scale is a 3-part, clinician-administered scale that assesses global illness severity and change. For inclusion criteria and to examine the effects of lanicemine, the first part, Severity of Illness is used. The CGI-S assesses overall severity of illness on a 1 to 7 point scale with 1 indicating "normal, not ill" and 7 indicating "among the most extremely ill patients". The CGI-I assesses global improvement on a 1-to-7 point scale with 1 indicating "very much improved," 4 indicating "no change" and 7 indicating "very much worse."

The **PTSD Checklist for DSM-5** (PCL-5) (12–14) is a 20-item self-report measure that assesses the 20 DSM-5 symptoms of PTSD. The PCL-5 shares similar reliability with the CAPS-5 and has a variety of purposes, including: screening for PTSD symptoms, diagnosing PTSD and monitoring symptom change during and after treatment.

Secondary Outcome Measures: Measures of Cross-sensitization

Historical features potentially associated with cross-sensitization are documented at intake, and include:

The **Mini-International Neuropsychiatric Interview** (MINI) (15) version 7.0.0, which is compatible with DSM-5 criteria, is a structured diagnostic interview to assess age of onset and severity of any psychiatric disorder, and the number of mood and PTSD episodes.

The **Childhood Trauma Questionnaire** (CTQ) (16) to assess childhood physical abuse, sexual abuse, emotional abuse, physical neglect, and emotional neglect.

The frequency and severity of substance use with the **Fagerstrom Test for Nicotine Dependence** (FTND) (17), a 6-item self-report measure assessing the intensity of physical addiction to nicotine.

The **Alcohol Use Disorders Identification Test** (AUDIT) Self-Report Version(18), a 10-item self-report scale to identify hazardous drinkers and to detect active alcohol use disorders.

The **Timeline Followback** (TLFB) (19) quantifies substance use.

The **Columbia-Suicide Severity Rating Scale** (C-SSRS) (20) to assess history of suicidal behaviors and ideations, including lethality of attempts and frequency, duration, controllability, reasons for ideation.

The **Quick Inventory of Depressive Symptoms-Self Report** (QIDS-SR) (21), a 16-item self-report depression inventory, assesses effects of lanicemine on depression symptoms.

DRUG PREPARATION AND RANDOMIZATION

Lanicemine will be prepared as a 1.0 mg/mL (100 mg dose) solution ready for infusion. Matching placebo is a 0.9% saline solution ready for infusion. The lanicemine infusion will be a final volume of 100

mL given at an infusion rate of 1.67 mL/min (1.667 mg/min) over 60 minutes. The placebo infusion will be a final volume of 100 mL given at an infusion rate of 1.67 mL/min over 60 minutes. Randomization is performed by the research pharmacist who has no patient contact, using a computer-generated random-number list that is based on a permuted block procedure. Only the pharmacist has access to the randomization code; clinicians, raters, and data analysts are masked to treatment group.

INCLUSION AND EXCLUSION CRITERIA

Inclusion Criteria

1. Male and female patients aged 21 to 65 years, inclusive.
2. CAPS-5 score ≥ 25 and CGI-S ≥ 4 at Screening and Randomization.
3. Anxiety Potentiated Startle T-score ≥ 2.8 at Screening and Randomization.
4. Psychotropic medications must remain at a stable dose for at least 42 days prior to screening, without clinically significant adjustment.

Exclusion Criteria

1. Patients who are currently participating in another clinical study in which the patient is exposed to an investigational or non-investigational drug or device, or have done so within the last 30 days prior to screening.
2. Patients with a history of DSM-5 diagnosis of bipolar disorder, schizophrenia or schizoaffective disorder, or currently exhibiting psychotic symptoms associated with depression; dementia or suspicion thereof, is also exclusionary.
3. Currently being treated with MAOIs, lithium, divalproex, carbamazepine, barbiturates, or benzodiazepines. Patients taking medications with known activity at the NMDA or AMPA glutamate receptor [eg, riluzole, amantadine, lamotrigine, memantine, topiramate, dextromethorphan, D-cycloserine], or the mu-opioid receptor.
4. Patients who meet DSM-5 criteria for substance use disorder (amphetamines, cocaine, hallucinogens, inhalants, opioids, sedatives/hypnotics/anxiolytics) within 1 month prior to screening. Patients with a positive urine drug screen (UDS) are excluded except for patients testing positive for prescribed medications that are otherwise permitted and there is no evidence of misuse or abuse. Patients can be re-tested only if either the initial opiate or barbiturate result is positive and they have a prescription, but the patient should be excluded if the result is still positive at the second test. Patients with positive UDS for drug(s) legally available by prescription must provide evidence of prescription for the drug(s).
5. Patients with a binge-pattern of alcohol use which makes them at risk for withdrawal-related seizures. Also, significant alcohol withdrawal symptoms that require medical detoxification.
6. Patients with a suicide attempt within the last 3 months or at imminent risk of suicide and not suitable for an outpatient study, in the judgment of the investigator.
7. Patients who are pregnant or lactating.
8. Clinically significant laboratory value, physical examination, or ECG that signifies a major medical illness that is unstable or inadequately controlled, or that may put the subject at risk during the study in the judgment of the investigator. Hypothyroidism is permitted if corrected and the patient is on a stable treatment regimen for a minimum of 6 months.
9. Systolic BP < 85 or > 160 mmHg or diastolic BP > 100 mmHg or heart rate < 50 or > 105 beats per minute at Screening or Randomization. Exclusionary values may be repeated once.
10. Patients with any history of seizure disorder (except for febrile seizures in childhood) or traumatic brain injury.

11. Previous exposure to lanicemine.
12. Patients with a personality disorder which in the opinion of the investigator has a major impact on the patient's current psychiatric status and would preclude safe study participation.
13. Body mass index ≥ 40 kg/m^2

REFERENCES

1. Schmitz A, Grillon C. Assessing fear and anxiety in humans using the threat of predictable and unpredictable aversive events (the NPU-threat test). *Nat Protoc* (2015) **7**:527–532. doi:10.1038/nprot.2012.001

2. Grillon C, Pine DS, Lissek S, Rabin S, Bonne O, Vythilingam M. Increased anxiety during anticipation of unpredictable aversive stimuli in posttraumatic stress disorder but not in generalized anxiety disorder. *Biol Psychiatry* (2009) **66**:47–53. doi:10.1016/j.biopsych.2008.12.028

3. Lieberman L, Stevens ES, Funkhouser CJ, Weinberg A, Sarapas C, Huggins AA, Shankman SA. How many blinks are necessary for a reliable startle response? A test using the NPU-threat task. *Int J Psychophysiol* (2017) **114**:24–30. doi:10.1016/j.ijpsycho.2017.01.012

4. Duncan CC, Barry RJ, Connolly JF, Fischer C, Michie PT, Näätänen R, Polich J, Reinvang I, Van Petten C. Event-related potentials in clinical research: guidelines for eliciting, recording, and quantifying mismatch negativity, P300, and N400. *Clin Neurophysiol* (2009) **120**:1883–1908. doi:10.1016/j.clinph.2009.07.045

5. Lijffijt M, Lane SD, Meier SL, Boutros NN, Burroughs S, Steinberg JL, Gerard Moeller F, Swann AC. P50, N100, and P200 sensory gating: relationships with behavioral inhibition, attention, and working memory. *Psychophysiology* (2009) **46**:1059–1068. doi:10.1111/j.1469-8986.2009.00845.x

6. Lijffijt M, Moeller FG, Boutros NN, Burroughs S, Lane SD, Steinberg JL, Swann AC. The role of age, gender, education, and intelligence in P50, N100, and P200 auditory sensory gating. *J Psychophysiol* (2009) **23**:52–62. doi:10.1027/0269-8803.23.2.52

7. Weathers FW, Blake D, Schnurr PP, Kaloupek DG, Marx BP, Keane TM. *The Life Events Checklist for DSM-5 (LEC-5) – Standard.* (2013). Available at: https://www.ptsd.va.gov/

8. Weathers FW, Bovin MJ, Lee DJ, Sloan DM, Schnurr PP, Kaloupek DG, Keane TM, Marx BP. The Clinician-Administered PTSD Scale for DSM-5 (CAPS-5): development and initial psychometric evaluation in military veterans. *Psychol Assess* (2018) **30**:383–395. doi:10.1037/pas0000486

9. Weathers FW, Blake DD, Schnurr PP, Kaloupek DG, Marx BP, Keane TM. *The Clinician-Administered PTSD Scale for DSM-5 (CAPS-5) – Past Month / Worst Month*. (2015). Available at: www.ptsd.va.gov

10. Weathers FW, Blake DD, Schnurr PP, Kaloupek DG, Marx BP, Keane TM. *The Clinician-Administered PTSD Scale for DSM-5 (CAPS-5) – Past Week*. (2015). Available at: www.ptsd.va.gov

11. Guy W. *ECDEU assessment manual for psychopharmacology*. Rockville, MD: US Department of Heath, Education, and Welfare Public Health Service Alcohol, Drug Abuse, and Mental Health Administration (1976).

12. Blevins CA, Weathers FW, Davis MT, Witte TK, Domino JL. The Posttraumatic Stress Disorder Checklist for DSM-5 (PCL-5): development and initial psychometric evaluation. *J Trauma Stress* (2015) **28**:489–498. doi:10.1002/jts.22059

13. Bovin MJ, Marx BP, Weathers FW, Gallagher MW, Rodriguez P, Schnurr PP, Keane TM. Psychometric properties of the PTSD Checklist for Diagnostic and Statistical Manual of Mental Disorders-Fifth Edition (PCL-5) in veterans. *Psychol Assess* (2016) **28**:1379–1391. doi:10.1037/pas0000254

14. Weathers FW, Litz BT, Keane TM, Palmieri PA, Marx BP, Schnurr PP. *The PTSD Checklist for DSM-5 (PCL-5)*. (2013). Available at: www.ptsd.va.gov

15. Sheehan DV, Lecrubier Y, Sheehan KH, Amorim P, Janavs J, Weiller E, Hergueta T, Baker R, Dunbar GC. The Mini-International Neuropsychiatric Interview (M.I.N.I.): the development and validation of a structured diagnostic psychiatric interview for DSM-IV and ICD-10. *J Clin Psychiatry* (1998) **59 Suppl 20**:22–33.

16. Bernstein DP, Fink L. *Childhood Trauma Questionnaire: a retrospective self-report; manual*. San Antonio, TX: Harcourt & Companyi (1998).

17. Heatherton TF, Kozlowski LT, Frecker RC, Fagerström KO. The Fagerström Test for Nicotine Dependence: a revision of the Fagerström Tolerance Questionnaire. *Br J Addict* (1991) **86**:1119–1127.

18. Saunders JB, Aasland OG, Babor TF, de la Fuente JR, Grant M. Development of the Alcohol Use Disorders Identification Test (AUDIT): WHO collaborative project on early detection of persons with harmful alcohol consumption--II. *Addiction* (1993) **88**:791–804.

19. Sobell LC, Sobell MB. *Alcohol Timeline Followback users' manual*. Toronto, Canada: Addiction Research Foundation (1995).

20. Posner K, Brown GK, Stanley B, Brent DA, Yershova KV, Oquendo MA, Currier GW, Melvin GA, Greenhill L, Shen S, et al. The Columbia-Suicide Severity Rating Scale: initial validity and internal consistency findings from three multisite studies with adolescents and adults. *Am J Psychiatry* (2011) **168**:1266–1277. doi:10.1176/appi.ajp.2011.10111704

21. Rush AJ, Trivedi MH, Ibrahim HM, Carmody TJ, Arnow B, Klein DN, Markowitz JC, Ninan PT, Kornstein S, Manber R, et al. The 16-Item Quick Inventory of Depressive Symptomatology (QIDS), clinician rating (QIDS-C), and self-report (QIDS-SR): a psychometric evaluation in patients with chronic major depression. *Biol Psychiatry* (2003) **54**:573–583.
